# Supplementary material for: Causal Relationship between Aging and Anorexia Nervosa: A White-Matter-Microstructure-Mediated Mendelian Randomization Analysis
Source: Biomedicines. 2024 Aug 16;12(8):1874. doi: 10.3390/biomedicines12081874 (PMC11351342; doi:10.3390/biomedicines12081874)
Supplement: Supplementary file 1 [file biomedicines-12-01874-s001.zip › Supplementary Figure/Figure S4/Singe SNP plot of MR analysis for TL to ubm-b-1822.pdf]

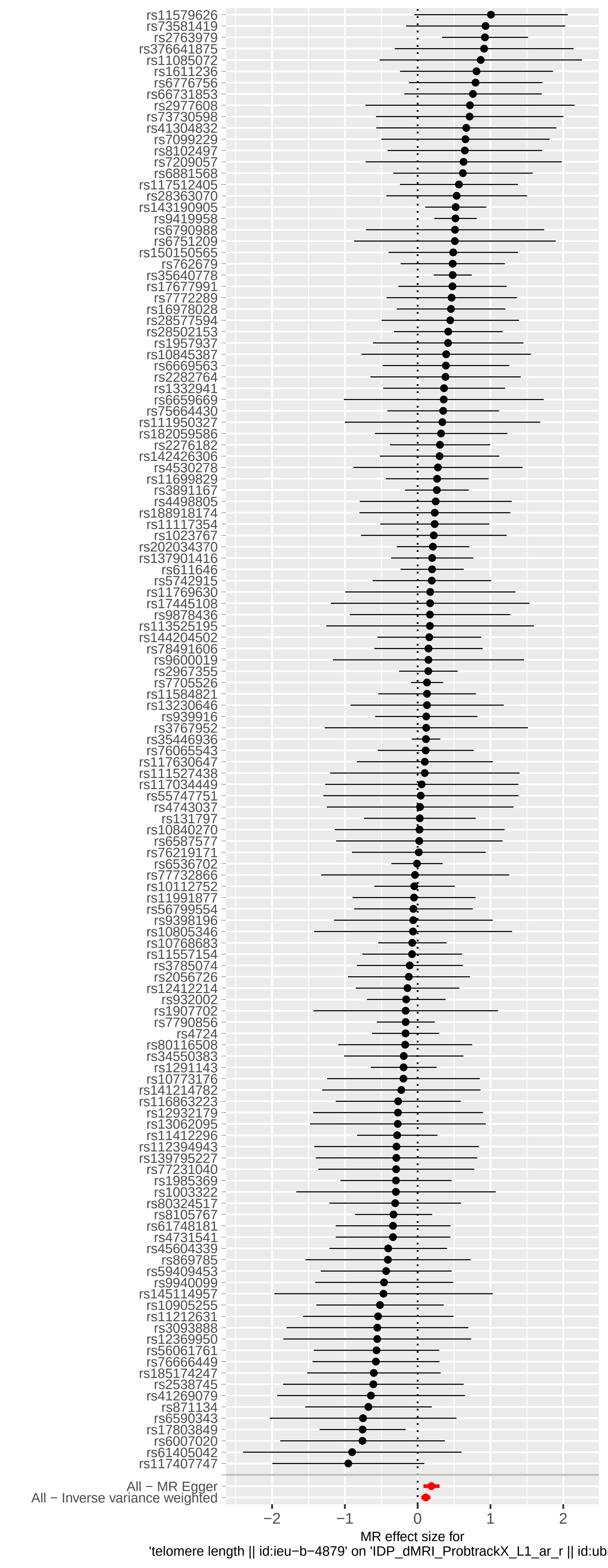
rs11579626
rs73581419
rs2763979
rs376641875
rs11085072
rs1611236
rs6776756
rs66731853
rs2977608
rs73730598
rs41304832
rs7099229
rs8102497
rs7209057
rs6881568
rs117512405
rs28363070
rs143190905
rs9419958
rs6790988
rs6751209
rs150150565
rs762679
rs35640778
rs17677991
rs7772289
rs16978028
rs28577594
rs28502153
rs1957937
rs10845387
rs6669563
rs2282764
rs1332941
rs6659669
rs75664430
rs111950327
rs182059586
rs2276182
rs142426306
rs4530278
rs11699829
rs3891167
rs4498805
rs188918174
rs11117354
rs1023767
rs202034370
rs137901416
rs611646
rs5742915
rs11769630
rs17445108
rs9878436
rs113525195
rs144204502
rs78491606
rs9600019
rs2967355
rs7705526
rs11584821
rs13230646
rs939916
rs3767952
rs35446936
rs76065543
rs117630647
rs111527438
rs117034449
rs55747751
rs4743037
rs131797
rs10840270
rs6587577
rs76219171
rs6536702
rs77732866
rs10112752
rs11991877
rs56799554
rs9398196
rs10805346
rs10768683
rs11557154
rs3785074
rs2056726
rs12412214
rs932002
rs1907702
rs7790856
rs4724
rs80116508
rs34550383
rs1291143
rs10773176
rs141214782
rs116863223
rs12932179
rs13062095
rs11412296
rs112394943
rs139795227
rs77231040
rs1985369
rs1003322
rs80324517
rs8105767
rs61748181
rs4731541
rs45604339
rs869785
rs59409453
rs9940099
rs145114957
rs10905255
rs11212631
rs3093888
rs12369950
rs56061761
rs76666449
rs185174247
rs2538745
rs41269079
rs871134
rs6590343
rs17803849
rs6007020
rs61405042
rs117407747
All − MR Egger
All − Inverse variance weighted
−2
−1
0
1
2
MR effect size for
'telomere length || id:ieu−b−4879' on 'IDP_dMRI_ProbtrackX_L1_ar_r || id:ub
